# Requirements for the Development of Bacillus Anthracis Spore Reference Materials Used to Test Detection Systems

**DOI:** 10.6028/jres.111.017

**Published:** 2006-06-01

**Authors:** Jamie L. Almeida, Lili Wang, Jayne B. Morrow, Kenneth D. Cole

**Affiliations:** Biochemical Science Division, Chemical Science and Technology Laboratory, National Institute of Standards and Technology, Gaithersburg, MD 20899-8610

**Keywords:** anthrax, *Bacillus anthracis*, bacteria, biological threat, detection, reference materials, spores

## Abstract

*Bacillus anthracis* spores have been used as biological weapons and the possibility of their further use requires surveillance systems that can accurately and reliably detect their presence in the environment. These systems must collect samples from a variety of matrices, process the samples, and detect the spores. The processing of the sample may include removal of inhibitors, concentration of the target, and extraction of the target in a form suitable for detection. Suitable reference materials will allow the testing of each of these steps to determine the sensitivity and specificity of the detection systems. The development of uniform and well-characterized reference materials will allow the comparison of different devices and technologies as well as assure the continued performance of detection systems. This paper discusses the special requirements of reference materials for *Bacillus anthracis* spores that could be used for testing detection systems. The detection of *Bacillus anthracis* spores is based on recognition of specific characteristics (markers) on either the spore surface or in the nucleic acids (DNA). We have reviewed the specific markers and their relevance to characterization of reference materials. We have also included the approach for the characterization of candidate reference materials that we are developing at the NIST laboratories. Additional applications of spore reference materials would include testing sporicidal treatments, techniques for sampling the environment, and remediation of spore-contaminated environments.

## 1. Introduction

The timely and reliable detection of biological threats is essential to secure the health and security of our nation. Biological threats come in many forms and in some cases, just a few organisms can replicate in the host to cause disease. The detection of biological threats is a multi-stage process and each stage must function efficiently for a surveillance system to be successful. The stages of a system include: the sample collection (interface to the environment), the sample preparation (into a form suitable for detection), and a specific detection step. Detection systems may either simply indicate the presence of a threat or quantitatively measure the concentration of the threat. Reference materials can serve as positive controls and standards for these systems. Each stage of the detection systems needs to be tested for a detection system to yield reliable and useful results.

The spores of *Bacillus anthracis* (BA) are particularly dangerous because they persist in the environment, and relatively small numbers can cause death. In addition, research has been done to prepare spore samples so that they are readily dispersed in the environment [1]. It is a difficult challenge to discriminate BA spores and vegetative cells from closely related bacteria (referred to as near neighbors). The specificity of detectors is the ability to distinguish between a real threat and non-threats, such as near neighbors. Another significant challenge is to be able to detect a real threat that may come in different forms. Spores may be processed to change their surface characteristics or added to different materials (matrices) that can significantly effect their detection with some devices and instruments. Testing the specificity and the effect of the form and matrix on the performance of a detector is essential to have confidence in the results obtained with real world samples. Such testing is a major study involving many samples and matrices. This is not the goal of the reference materials we are developing. Our goal is to provide a reference material that can be used as a material with uniform and measured properties to test the proper functioning of detectors and calibrate laboratory instruments.

The availability of uniform and well-characterized reference materials will aid in the development of new surveillance systems, allow meaningful comparisons of existing systems and ensure that existing systems are being used and are operating in the proper manner. This paper will focus on the unique properties of spore materials that need to be considered for their development as reference materials. An accompanying paper will cover the properties that will be used to characterize candidate reference materials for another class of biological threats, which is the protein toxin ricin (submitted).

The detection of BA spores is based on recognition of specific characteristics (markers) on either the spore surface or in the spore nucleic acid (DNA). We will review the use of these markers for detection of BA and their relevance to characterization of reference materials. This paper considers the special requirements for the development of reference materials for BA spore detection systems and the approach to characterize these materials in the NIST laboratories.

## 2. Biochemical Markers of BA Spores

### 2.1 Spore Structure

Some species of bacteria form spores to survive unfavorable environmental conditions. Bacterial spores sense their environment and when conditions are again favorable, they will revive in a process termed germination. The life cycle of spore-forming bacteria consists of stages of vegetative growth, sporulation, germination, and outgrowth [2–4]. When the vegetative cells are committed to sporulation by environmental cues, the mother cell (the sporangium) contributes the complex layers of the spore coats that encase the spore DNA. The mother cell dies and begins to fall apart at the end of sporulation, and this contributes cellular debris containing vegetative cell proteins, carbohydrates, and DNA. Crude (unprocessed) spore preparations will contain large amounts of materials derived from the mother cells as well as culture media used to grow the cells, in addition to the spores.

Bacillus spores contain a number of coat layers and some species posses an additional outermost layer called the exosporium. BA, *B. cereus*, and *B. thuringiensis*, have all been observed to have an exosporium. These three species are closely related and make up what is known as the *B. cereus* group, and some argue that they should be classified as the same species in which some members have acquired additional virulence factors [5, 6]. Since the exosporium is the outermost layer of the BA spores, it likely contains important protein and carbohydrate markers that are recognized by antibodies and other molecular recognition molecules [7, 8]. Imaging using atomic force microscopy has revealed the details of the exosporium under dry and fully hydrated conditions [9]. The exosporium appeared as a large envelope (25 nm to 40 nm thick) surrounding the spore with hair-like projections and additional tubular appendages [9]. The exosporium of *Bacillus cereus* is composed of about 50 % proteins, along with lower amounts of lipids and carbohydrates [10].

Spores, are oval shaped with dimensions of approximately 1.5 µm × 1 µm (Fig. 1), and are relatively resistance to inactivation by heat and irradiation. The spores are refractive to light and under phase contrast microscopy appear bright under these conditions.

### 2.2 Proteomic Approaches to Identify Spore Markers

New approaches based on proteomics have the promise to develop additional spore specific markers for BA detection. Proteomic analysis using two-dimensional gels and mass spectroscopy of samples from BA, and the close relative *B. subtilis*, has identified a number of spore coat proteins [11] and proteins associated with germination [12]. A comprehensive proteomics and gene expression approach was used to examine the process of sporulation of BA (Sterne strain) [13]. This study identified over 750 proteins present in the spores. Subcellular fractionation of the spores identified a number of proteins and genes associate with the exosporium [13, 14]. An observation of this study was that some proteins associated with the exosporium could be removed by water washes and they may be fortuitously bound to the exosporium [13].

Protein analysis of the exosporium has yielded several proteins with enzymatic activity [15]. A collagen-like glycoprotein (BclA) has been identified as a component of the hair-like layer of the BA exosporium [16, 17]. BclA was shown to be the immunodominant protein on the surface of BA spores [16]. BclA has a central repeating tri-peptide unit, and the length varies with the strain of BA [16, 17]. The polymorphisms in the BclA protein in different strains of BA were correlated to changes in length of the hair-like filaments of the exosporium layer of the spores [18]. The structure of two O-linked oligosaccharides of BclA has recently been determined [19].

The complete genomic sequences of the BA chromosome and the plasmids have been determined [20]. The genomic data combined with proteomic approaches hold the promise to determine the total protein complement of the complex structures of the spore coats. Longer-term goals would be to determine the structure and biological role of the spore coats. New approaches to develop affinity ligands based on peptides have been developed that specifically bind to the surfaces of BA spores and may improve the specificity of detectors for BA [8].

### 2.3 Surface Markers for Vegetative BA Cells

The classical methods to confirm the presence of BA include culturing of samples on plates and immunological detection of capsule formation and the susceptibility to lysis by gamma phage [21]. An additional confirmatory test for the presence of BA is analysis of the distinctive fatty acid methyl esters in vegetative BA cells [22]. These methods are specific for the vegetative form of BA and are not used to detect spores directly, only after they have germinated and have been grown on culture media to form vegetative cells. The specific detection of vegetative cells of BA cells or fragments of dead cells will require specific markers. Antibodies were found against two BA vegetative cell proteins termed EA1 and EA2 (extractable antigens) in the serum of guinea pigs that were vaccinated with BA Sterne vaccine preparations [23].EA1 had a mass of 91 kilodaltons (kDa) and appeared to be coded for by the chromosome and EA2 with a mass of 62 kDa appeared to be coded for by a gene on the pXO1 plasmid [23]. A BA chromosomal gene coding for protein termed S-layer protein was isolated and characterized [24]. The S-layer is an ordered array of proteins on the surface of some bacteria. The S-layer protein has a calculated molecular weight of 83.7 kDa, but appeared higher (94 kDa) on SDS PAGE and as the authors speculate the S-layer protein may be the same as EA1 [24].

### 2.4 Spore Surface Properties and Interaction Forces

Spore surface features can alter surface properties, including charge and hydrophobicity, which can have a profound impact on detection and fate in the environment. The size and shape of bacterial spores makes them susceptible to microscopic interaction forces (van der Waals, electrostatic, and hydrophobic) that influence spore fate and persistence in the environment. The relative magnitude of such microscopic interaction forces is dependent on the ionic strength of the suspending solution and the surface chemistry of the interacting surfaces. Changing the relative hydrophobicity or altering the solution's ionic strength provides a means to alter environmental fate of bacteria and bacterial spores. For example, weapons grade spores are modified with silica particles altering the electrostatic repulsion between spores therefore optimizing atmospheric dispersion [1]. Surface hydrophobicity is known to contribute to cell aggregation (clumping) and air-water partitioning by counteracting electrostatic forces which are repulsive for like charged surfaces in most environmentally relevant fluids [25, 26]. Hydrophobic spores were less susceptible to UV disinfection in drinking water treatment by increasing spore clump formation [27]. Such hydrophobic interactions have been shown to impact the fate of potentially pathogenic bacteria in groundwater [28] and drinking water systems [29]. Expression of an exosporium, a trait of BA, *B. cereus*, and *B. thuringiensis*, results in a more hydrophobic spore surface than the weaker hydrophobic component measured for the spore coat [30].

Purification and storage treatments can have a significant impact on the surface properties of bacterial spore suspensions. Lysozyme and protease treatments are commonly used to remove outer spore coat proteins in methods to isolate spore DNA. Treatment with enzymes or detergents can potentially remove surface markers that will effect the detection of the spores. Although data directly on BA spores surface characteristics and surface stability may be not available, insight into the problems may be gained from studies using other species. Banding bacterial spores in density gradients is commonly used to purify spores, but the choice of gradient material can influence the measured density of the spores presumably due to partial permeation of the spores by the medium used [31]. Heat treatment has been shown to increase the relative hydrophobicity of spores [32]. Additionally, long-term storage at temperatures ranging from −80 ºC to 4 ºC resulted in significant changes in fungal spore germination rates due to ultrastructural changes [33]. Alteration of surface properties by chosen purification and storage methods must be addressed when determining optimal conditions for manufacture of a standard BA spore preparation. The choice of purification method may influence the stability of the spores, as seen in other *bacillus species* [34].

### 2.5 Virulence Factors, Plasmid DNA Markers, and Assays

The virulence factors of BA include the anthrax toxin and the poly-D-glutamic acid capsule that surrounds vegetative cells. The anthrax toxin has three components including edema factor, lethal factor, and protective antigen. The pXO1 plasmid from BA (Sterne) (181.7 kilobase pairs (kbp)) was sequenced and found to contain 143 possible genes including the three toxin components [35]. The gene for the protective antigen (*pag*) was sequenced in a number of strains of BA and five point mutations were identified allowing classification of BA strains into six genotypes [36].

Genes that code for a poly-D-glutamic acid capsule are found on the pXO2 plasmid in BA. The pXO2 plasmid contains the genes *capB*, *capC*, and *capA* that are three membrane enzymes that are required for the capsule of BA cells [37–39].

PCR primers for *capA* have been used for PCR to identify BA [40].A quantitative PCR (QPCR) assay for detection of BA was developed using the *cap* and *pag* genes as markers [41–43]. Lief et al. (1994) used the *capB* gene as a target to detect BA using PCR followed by hybridization of two labeled probes. The complex was captured and measured using a novel method based on the activity of urease enzyme [44].A rapid QPCR assay using the *cap* gene as a target for BA detection used internal DNA controls [45]. The internal control sequences were designed to have a guanine cytosine base pair content that would allow the determination of the control from the BA target by differences in the melting temperature of the PCR products [45].

*B. cereus* (American Type Culture Collection #10987) DNA was sequenced and found to be closely related to BA and contained a large plasmid (208 kbp) that was similar to pXO1, but did not contain the lethal factor and edema toxin genes [5].

QPCR was used to measure the copy numbers of pXO1 and pXO2 plasmids and a BA chromosomal marker (Ba813) in vegetative cells. [46]. They found that, when DNA was isolated from vegetative colonies, high copy numbers of the plasmids pXO1 and pXO2 were measured [46].

### 2.6 Chromosomal DNA Markers and Assays

Sequencing of the 16S rRNA gene of BA and near neighbors confirmed the close relationship of the *B. cereus* group, but a few single basepair changes could be used to distinguish BA from the strains examined [47]. The complete sequence of the BA chromosome (5.23 mega-basepairs) has been determined from the Ames strain [20]. Comparative hybridization of DNA from *B. cereus* and *B. thuringiensis* using microarray analysis also showed the close relation of BA to these species [20]. The marker *vrrA* (variable region with repetitive sequence) was used to distinguish between different strains of BA and near neighbors [48]. The chromosomal marker *gyrA* (coding a part of the DNA gyrase enzyme) has been used to develop a quantitative PCR (QPCR) assay that was specific for BA [49]. A randomly amplified polymorphic DNA (RAPD) method was used to select a marker (SG-850) that could be used to identify members of the *B. cereus* group of bacteria including BA in this group [50]. Multiple-locus variable-number tandem repeat analysis (VNTR) was developed and used to type and deduce relationships of different strains of BA [51]. A number of markers including single nucleotide polymorphisms (SNP), nucleotide inserts, deletions, and tandem repeats have been developed [52]. Recently mass spectroscopy has been applied to VNTR and SNP analysis of BA isolates [53].

Additional chromosomal markers have been used to identify BA using PCR, including Ba813 [54, 55]. The occurrence of Ba813 was also found in some strains of *Bacillus cereus and Bacillus thuringiensis* [55, 56]. The Ba813 marker has been used for a real time PCR assay using Taqman-type probes to measure BA in clinical samples and powders [57]. The *rpoB* gene codes for the beta-subunit of RNA polymerase and has been used to identify phylogenetic relationships among bacteria [58]. A real-time PCR assay was developed for BA using the *rpoB* gene as a chromosomal marker [59]. Qi et al. [59] used fluorescence resonance energy transfer (FRET) type probes for a real-time PCR assay. This assay was used to detect BA in clinical samples [43]. The *rpoB* FRET probes were combined with primers and FRET probes for *pag A* (pXO1) and *capC* (pXO2) markers to increase the confidence in detecting virulent BA strains [60].

Ellerbrok et al. [61] developed Taq-Man type QPCR assays for the chromosomal marker (*rpoB*), a marker for pXO1 plasmid (*pag*), and a marker for pXO2 plasmid (*capC*). A multiplex PCR assay based on *rpoB* was used to identify strains of BA [62]. Levi et al [63] developed a multiplex PCR assay using universal bacterial 16S rDNA primers (for a positive control), Ba813 primers [54], and a trans-activator of encapsulation marker for the gene *acpA* [64]. The *sap* gene that codes for the structural protein of the S-layer of BA has been characterized [24]. The *sap* gene has been used as a target for PCR detection of BA in meat [65]. A QPCR assay used for the detection of BA from soil utilized Taq-man probes for *sap gen*e, the *pag gene*, and *cap gene* [66]. A DNA subtraction strategy was used to isolate a chromosomal DNA sequence (clone B26) that was highly specific for BA species [67]. This sequence was used to develop a real time PCR assay [67].

The Laboratory Response Network of the Centers for Disease Control and Prevention uses a real-time PCR assay with probes for both plasmids and a chromosomal marker [68]. This assay is used to confirm BA from vegetative colonies from plates and spore samples. The approach of using three markers (for each plasmid and chromosome) increases the confidence of the assay results detecting virulent forms of BA.

### 2.7 DNA Extraction From Spores for Detection

The detection of the markers based on DNA from either the chromosome or plasmid requires their release from the spore. This is the sample preparation step that is critical for the success of detection methods using DNA. Luna et al. (2003) made a study of the effect of various treatments on the detection limit of BA using quantitative PCR with the Ba813 chromosomal marker [57]. They examined the effect of heat shock, sonication, and autoclaving on the detection of the spore DNA. When the spores were heat shocked, sonicated, and autoclaved, and the liberated DNA was concentrated, less than 10 spores could be detected in a sample [57]. Mechanical breaking of spores using processes such as bead beating released DNA in a form suitable for PCR assays [69]. Mechanical treatments have the advantages that they do not depend upon enzymatic treatment or a biological response (germination) for detection.

Ellerbrok et al. (2002) were able to detect signals in QPRC from 800 spores directly in a QPCR reaction mixture with 20 % efficiency [61]. When BA spore samples were germinated and incubated in culture media for up to 6 h, they saw a large increase in the efficiency of detection, although during this time germination and replication may have taken place [61]. Rief et al. (1994) examined the effect of adding spores directly to a PCR mixture, germination of the spores, and mechanical disruption (bead-beating) for detection of BA [44]. They found that germination and mechanical disruption increased the release of DNA from the spores, and that adding spores to the PCR mixture was not effective. Their results indicated that the DNA detected from spore preparations with just heat treatment came from extracellular sources [44].

Dang et al. (2001) compared the effect of gamma irradiation and autoclaving on the detection of BA spores by antibody detection and PCR detection [70]. The effect of irradiation and heat-treatment on detection limits could be increased or decreased dependent upon the antibody preparation. Irradiation and heat-treatment of the BA spores resulted in a decrease in the fluorescence signals of real-time PCR assays [70]. Several studies have examined the effect of inactivation by heat of BA on the detection using PCR. Fasanella et al. (2003) autoclaved (121 ºC for 45 min) freeze-dried BA spores and compared the results to control spore samples (heated 98 ºC for 30 min), and found a decrease in the detection limit of 10-fold [71]. Differences in the form of the spores (powder vs liquid suspension) that are inactivated could likely have an effect on the detection efficiency.

## 3. Approaches to Measurement of Spore Properties for Reference Materials

### 3.1 Introduction

Table 1 shows the properties and some potential measurement techniques that could be used to characterize the BA spore reference materials. The use and limitations of each measurement technique will be discussed based on our current knowledge. An important consideration of the spore reference material is the source and form of the materials. The reference materials should meet as many of the needs for testing detection systems as is practical. Reference materials have the requirements of homogeneity and stability. Ideally, the materials should be safe to use and not constitute a security risk.

### 3.2 Choice of the Strain of *Bacillus Anthracis*

The reference materials we intend to develop will be useful as positive controls, standards for detection systems, and as well, to test remediation and sampling procedures. Ideally for these purposes, the materials should be as close to a “typical” spore preparation as what is likely to be encountered. The reference materials we are developing will provide a uniform and well-characterized material that will be useful for many applications. Our initial plan is to provide spore suspensions of a single strain of BA that would be produced in large quantities to provide continuity for the users. The reference materials are to be tested for the homogeneity and stability of the measured properties. A good model of this would be the Standard Reference Material Program of NIST.

Non-virulent and inactivated materials offer safety to operators and reduce the possibility of viable bacteria falling into the hands of those who should not posses select agents. BA strains that lack either both pXO1 and pXO2 plasmids (pXO1^−^, pXO2^−^) or those that lack pXO2 (pXO1^+^, pXO2^−^) are excluded from the select agent rules [72]. The Sterne strain of BA (pXO1^+^, pXO2^−^) is widely used as a live spore suspension in veterinary medicine to vaccinate livestock against anthrax [73, 74]. The spores of BA (Sterne) would be expected to behave in a similar manner to those of virulent forms of BA, since the genes on the pXO2 plasmid, responsible for capsule formation, are expressed only in the vegetative cells. A recent study compared the inactivation of *B. subtilis* spores to BA Sterne [75]. The authors stated for meaningful comparisons, it is important to control conditions of growth, sporulation, spore purification, irradiation, dosimetry, and survival determination [75]. When these conditions were met, BA Sterne and *Bacillus subtilis* had comparable inactivation kinetics.

The use of BA (Sterne) spores will provide spores with similar properties to other strains of BA, but it will lack any markers on the pXO2 plasmid. We are evaluating spores containing pXO2 plasmid rendered nonviable by either gamma irradiation or heat sterilization as potential sources of these markers. The stability of inactivated materials will have to be determined.

### 3.3 Measurements of Bioactivity

Determination of the virulence of a BA strain or preparation requires animal studies, specialized laboratories, and highly trained personnel. For these reasons, it is likely that virulence measurements will be limited to specialized studies comparing the properties of different BA strains [46]. The viability of a BA spore preparation is most commonly done by measuring colony formation on nutrient agar (plate counts). Plate counting yields the number of viable colonies and the results are called colony-forming units (cfu). For accurate quantitation, plate counting is dependent upon dispersion of the bacteria into single cells (or spores). A clump of spores regardless of size will result in a single colony, so the plate count can under estimate the total number of spores if clumps are present.

Figure 1 shows images from phase microscopy of two preparations of BA (Sterne) suspensions. The two preparations show obvious differences in the amount of vegetative debris observed and one of the preparations shows visible clumping of the spores, a condition that is likely to have a significant effect in their behavior in many detection systems and inactivation studies.

**Fig. 2:**
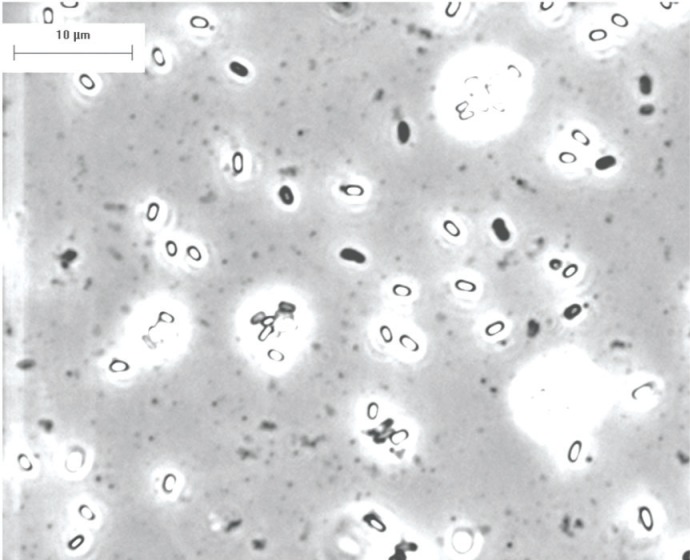
Phase microscopy of *Bacillus globigii* (*atrophaeus*) spore preparations.

Clumping of spores is a serious problem and additional work needs to be done on the conditions for preparation and storage that will minimize clumping. We have used a dried preparation of *B. globigii* (more recently classified as *B. atrophaeus*). Large clumps of this material are observed under phase microscopy (Fig. 2). Mechanical or chemical treatment (detergents or enzymes) of the spore suspensions has been proposed to reduce the level of clumping. These treatments to reduce clumping may be useful for analysis (to obtain a better plate count), but their use to prepare standards runs the risk of changing the nature of the materials and may interfere with some detection methods.

### 3.4 Measurements of Concentration

The BA spore detection methods target one or more specific marker(s) either on the surface of the spores or present in the nucleic acids, as previously discussed in Sec. 2. The determination of the bioactivity measures the concentration of viable spores, but many of the detection methods will detect their target, if present in a viable or non-viable form. This implies that dead spores or fragments will still be detected in those assays. Antibodies are prepared in animals that will vary in the immune response. Because of this antibody preparations will vary in their titer and specificity. Antibody specificity can be improved by the use of monoclonal antibodies and affinity purification of polyclonal preparations. In many cases, the total number of surface markers will not be defined and could possibly vary with the strain of BA. For these reasons, antibody assays are difficult to standardize in terms of sensitivity and specificity.

New PCR instruments (QPCR), based on the fluorescence detection of the DNA product (the amplicon), allow the quantitative determination of the concentration of the starting material. The accurate determination of starting concentration is dependent upon careful development of the assay and DNA standards. Coker et al. (2003) cloned the PCR products into plasmids and used purified plasmid DNA as standards. A series of plasmids were made containing sequences from the *capC* and BA813 markers that could be used in nested PCR assay as internal controls for BA detection [76]. Ellerbrok et al. (2002) used TaqMan-type QPCR assays for *rpoB*, *pag* (pXO1-specific), and *capC* (pXO2-specific) genes and cloned the PCR products into plasmids for use as standards [61].

We are developing a method to extract DNA from spores using chemical and enzymatic lysis of the spores. Measurement of the DNA extracted from the spores can be used to calculate genomic equivalents based on the molecular weight of the BA chromosome [20]. QPCR using chromosomal markers that are present at one copy per BA chromosome will yield genomic equivalents. We are doing the same measurements with the plasmid markers. The genomic equivalents determined by DNA analysis should correlate with the spore counts determined by plate counts and hemocytometer.

### 3.5 Measurements of Purity

The most likely contaminants of a spore preparation are the components derived from the vegetative cells and culture media. The processing of the spore preparation is likely to have a heat shock step designed to inactivate any surviving vegetative cells. The contamination of the spore preparation by vegetative components could be done by several approaches as outlined in Table 1. Quantitative image analysis of phase contrast images such as shown in Fig. 1, could be used to sort the visible fields into typical spore morphology, possible clumps, and debris. If antibodies of sufficient specificity were obtained it would be possible to use immunoassays to determine the concentration of vegetative markers present in the spore preparation. The content of DNA that is contributed by the vegetative cells (extra-spore sources) will be determined using QPCR markers as outlined above. The spores are designed by nature to persist in the environment and because of this are likely to be stable under the appropriate storage conditions. The contaminants derived from the sporulation process are derived from the process of cellular autolysis. Therefore the contaminants would not be expected to be stable. This will complicate the analysis of the contaminants. Because of this we are working to remove contaminants from the spore preparations using conditions that do not change the properties of the spores.

## 4. Conclusions and Additional Research

We are using the outline contained in Table 1 to guide our measurements for the development of BA spore reference materials. The materials we are focusing our efforts on are liquid suspensions of BA (Sterne). These materials appear to be able to fulfill all requirements for BA spore reference materials, with the exception of DNA markers for the pXO2 plasmid. To provide these markers, we are determining the suitability of inactivated BA spores (containing pXO2 genetic materials) as an additional supplement for pXO2 plasmid markers.

The relevant properties of the reference material must be stable for the life of the reference materials. Work done on different species of *bacillus* have indicated that the methods of preparation and purification used for the spores can change their properties and stability [34]. It is essential to do research on BA spore preparations to determine the methods that preserve the integrity and stability of the preparations. Research is needed to determine the optimal storage conditions and the useful life span of the reference materials.

We are currently developing protocols that would be used to minimally characterize the BA spore reference materials. Initially such measurements would be done on representative samples from a large batch of material and the stability of the measurements will be determined in a manner similar to those done to characterize NIST standard reference materials. The details of our analysis will be included with the data. Our initial plans are to include the following measurements as minimal to adequately characterize spore reference materials:

1. Colony forming units by plating before and after treatments to reduce clumping.
2. Spore count by hemocytometer before and after treatments to reduce clumping.
3. Microscopic image analysis of spore preparations.
4. Measurement of the intra-spore genomic equivalents using quantitative QPCR for at least one chromosomal marker and the plasmid markers.
5. Measurement of extra-spore DNA contents using above makers. This would be determined by measuring the DNA content of the preparation without any treatment that would liberate the DNA in spores.

The extra-spore material in the spore preparations is contributed by the death of the mother cells or dead spores that have released their contents. These extra- spore materials are not stabilized or protected within the spore coats. It would be reasonable to assume that the vegetative contamination would be more likely to degrade and change their concentrations. Because of this, it would be best to produce spore preparations with very low amounts of contaminants.

It would be essential to provide a marker for the pXO2 plasmid. One potential source of this would be inactivated spores from a virulent strain containing pXO2. We are currently working on establishing the utility and stability of such materials.

The clumping of spore preparations is a serious problem, not only to determine accurate counts of the spores, but they are also likely to behave differently in various detection and collection devices. The presence of clumps is also likely to have an effect on the inactivation kinetics by sporicidal treatments. Additional research is needed to prepare spore preparations with minimal clumping, low amounts of contaminants, and that are stable during storage.

One area that is related to the condition of spore clumping is the measurement of the surface characteristics of spores. The studies discussed above provide some evidence that spores have weak binding of proteins on their surface. Additional research is needed to confirm the binding and determine if it changes with the processing of the spores, storage conditions, and age of the preparation. The measurement of surface characteristics such as zeta potential (or charge measurements), hydrophobicity, and binding to well-characterized surfaces will improve our knowledge and provide insight into the behavior of spores in solution and adhesion to surfaces. Reports of low levels of germination in stored spore preparations [34], indicate the need to study storage conditions to hopefully reduce this process in order to increase the stability of the standards. Research should be done with spore preparations that have different properties (clumping, purity, etc.) to determine their effect on the performance in different detection, collection, spore disruption, and inactivation methods.

## Figures and Tables

**Fig. 1a:**
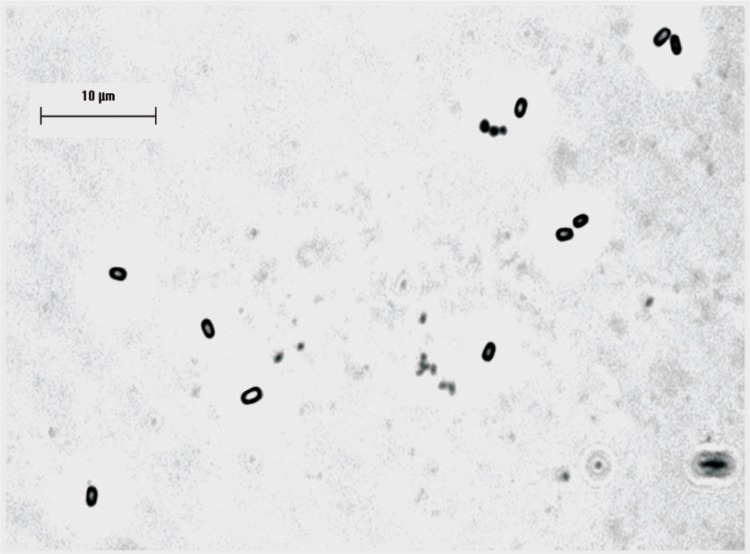
Phase contrast microscopy image of BA (Sterne) preparation. Bar is 10 µm.

**Fig. 1b:**
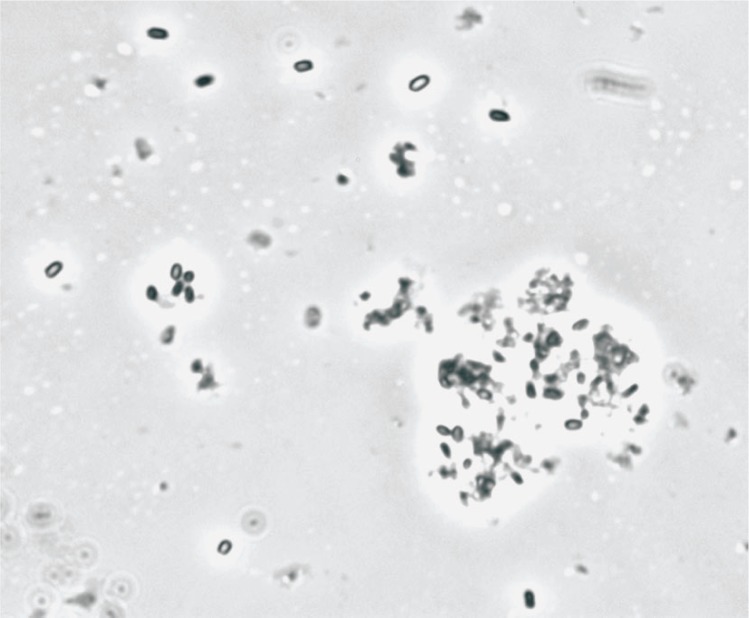
Phase contrast microscopy image of BA (Sterne) preparation.

**Table 1:** Properties and potential measurement techniques to characterize spore reference materials

Property	Measurement Technique	Barriers (Complicating factors)	Values
Bioactivity	Plating (viability)	Clumping	Colony forming units/mL
	Virulence	Animal studies	Lethal dose
Concentration	Hemocytometer	Clumping	Number of spores/mL
	Flow cytometer	Clumping, equipment, tags	Number of spores/mL
	Immunoassay	Antibody specificity	Target concentration
	DNA (after spore disruption)	Efficient release	Genomic (plasmid) equivalents/mL
Purity	Microscopic image analysis	Representative sampling	Ratio spores to debris
	DNA (without spore disruption)	Degradation of DNA	Extra-spore DNA/mL
	Immunoassay	Antibody specificity	Vegetative antigen concentration
